# Dr. Waldo and the Salvation Army

**Published:** 1895-11-30

**Authors:** 


### Dr. Waldo and the Salvation Army.

The victory which Dr. Waldo gained last week over the Salvation Army in the matter of the Blackfriars shelter is one on which he ought to be most cordially congratulated, not alone by his own personal friends, who have admired his pluck and perseverance in waging war against what he considered an evil and an abuse, but by all who look upon the Public Health Acts as seriously meant, and medical officers as seriously appointed for the protection of the public well-being.

By his decision in this case Mr. Slade has practically reduced the number to be received in the shelter in question by one half, for in issuing an order prohibiting overcrowding he fixed the limit of accommodation for the future at 550, whereas it appeared from the official figures that the numbers admitted had run so high as 1,045. The Salvation Army truly only contended that the buildings contained cubic space to accommodate 800 persons, but it must not be overlooked that one, if not more, of their witnesses maintained that even that number was quite within the mark, and that far more could be taken in without injury to health, so that we may feel quite sure that but for the energy of Dr. Waldo, and the common sense of the magistrate, the evils of these shelters would in the coming winter have been multiplied exceedingly.

The matter, however, cannot be said to be ended by a simple prohibition order. From the sanitary side it may well be asked what assurance is there that the limit now fixed will be adhered to; and how are the sanitary authorities, any more than before, to ascertain what is going on within these places, to which they have been refused admission except by a magistrate's order? From the philanthropic point of view also the question put by Mr Bramwell Booth is not without importance, viz., What is to be done with the 250 unfortunate and hungry men who have hitherto been able night after night to find at least shelter from the weather at the hands of the Salvation Army? In regard to this, what is of most importance is to know how far the running of these shelters is a commercial success. The law has in our opinion been sadly strained to allow these so-called charitable shelters to escape the restrictions enforced in the case of all common lodging houses and casual wards. The commercial question then becomes one of considerable interest, for if it can be shown that these places can be made to pay, and more especially if it can be shown that they do pay, the bottom will be knocked out of the sole reason or pretence for giving privileges to them which are not accorded to others in the trade.

It is by no means certain that, if the public chose to throw to the winds all the sanitary regulations which it has made by law for its own protection, the provision of a "penny doss" might not be a quite remunerative commercial enterprise (and then, of course, the difficulty to meet which the shelters were established would vanish), and we are not at all sure that, even if all sanitary requirements were honestly met, such an establishment could not be made to pay if worked on sufficiently large a scale. This, however, is not now the question. What the public is concerned about is that the same sanitary conditions which are enforced as a minimum both upon private individuals and public bodies, who make it their business to provide lodgings for the casual poor, shall also be made obligatory upon the Salvation Army, so far as they engage in the same line of business, whether the business pay or not and whether it be or be not a charity. Whatever multitude of sins charity may hitherto have covered, she certainly cannot be allowed to throw her cloak over defiance of sanitary law and the development of centres of infection in our midst. It is much to be regretted that Mr. Slade, in giving his decision last week, did not frankly accept the regulations already in force in regard to all analogous institutions; but, even as it stands, the decision is of considerable importance as showing that such bodies as the Salvation Army cannot be allowed to be a law unto themselves, and that if they infringe sanitary regulations they must, at the least, defend themselves before a magistrate. It would have been a sanitary disaster if this case had been dismissed, and the greatest credit is due to Dr. Waldo for his action in the matter. He has been none too well supported by his Vestry, while he has been abused for weeks past by "good" people, who believe in unregulated philanthropy. It would have been far easier for him to have let the whole thing drop, and it speaks well for his enthusiasm that he did not do so. Men of this stamp deserve promotion, for it is on the pluck and devotion of its medical officers of health that the country largely depends for its protection from sanitary dangers.

